# Use of data-mining to support real-world cost analyses: An example using HER2-positive breast cancer in Iran

**DOI:** 10.1371/journal.pone.0205079

**Published:** 2018-10-01

**Authors:** Amir Ansaripour, Kazem Zendehdel, Niki Tadayon, Fatemeh Sadeghi, Carin A. Uyl-de Groot, W. Ken Redekop

**Affiliations:** 1 Erasmus School of Health Policy and Management, Erasmus University Rotterdam, Rotterdam, The Netherlands; 2 Cancer Research Center, Cancer Institute of Iran, Tehran University of Medical Sciences, Tehran, Iran; 3 Department of General and Vascular Surgery, Shohadaye Tajrish Medical Center, Shahid Beheshti University of Medical Sciences, Tehran, Iran; University of Florida, UNITED STATES

## Abstract

**Introduction:**

Patient registries play an important role in obtaining real-world evidence of the cost-effectiveness of treatments. However, their implementation is costly and sometimes infeasible in many middle-income countries (MICs). We explored the combination of data-mining and a large claims database to estimate the direct healthcare costs of HER2-positive breast cancer (BC) treatment in Iran and the fraction of total costs from trastuzumab use.

**Method:**

We performed a retrospective analysis of claims data from the Iran Social Security Organization, a health insurer which covers approximately 50%(~40 million) of the Iranian population, in the period of 21/03/2011-20/03/2014. A data-mining algorithm using R software and validated using patient dossiers in the Cancer Research Center identified 1295 patients and divided them into the three main HER2-positive breast cancer stages (early, loco-regional and advanced). A payer perspective was used to calculate the absolute and relative direct costs of healthcare services associated with the treatment of HER2-positive breast cancer in the public and private healthcare systems.

**Results:**

The number of women totaled 802 (early), 125 (loco-regional) and 218 (advanced). The mean age[SD] was 45[10], 46[10] and 48[10] years, respectively, while mean follow-up in all stages was approximately one year. Average costs of direct healthcare care in early, loco-regional and advanced stages were €11,796 (95%CI: €9,356-€12,498), €8,253 (95%CI: €6,843-€10,002), and €17,742 (95%CI: €15,720-€19,505), respectively. Trastuzumab accounted for the largest share of total costs in all three stages (range: 53–76%).

**Conclusion:**

Wherever comprehensive patient registries are infeasible or costly, real-world costs can be estimated through claims databases and data-mining strategies. Using this method, real-world costs have been estimated in Iran. The stage-specific cost estimates derived from this study can be used to perform real-world cost-effectiveness analyses of therapies for HER2-positive BC and support healthcare financing decisions.

## Introduction

Useful cost-effectiveness analyses require sufficiently detailed data on both costs and health outcomes to support decisions about the reimbursement or implementation of new interventions [1]. Patient registries are an important source of patient level data that can be used to estimate the real-world costs and effectiveness of treatments. A good example is the Swedish National Patient Register which covers more than 99% of hospital services with a validation rate of 85–95% [2]. However, implementation of patient registries is costly and sometimes infeasible in many middle-income countries (MICs). Therefore, other strategies may be needed to obtain the data needed for a cost-effectiveness analysis. Claims databases are one of the possible sources to obtain valuable data on resource use in MICs. In this study, we describe how we used a claims database to obtain important stage-specific information on breast cancer (BC) in an MIC. Breast cancer was chosen since it is the most frequent cancer in women worldwide with an estimated 1.67 million new cancer cases diagnosed in 2012 (25% of all cancers)[3–5].

BC has become one of the most frequent malignancies among Iranian women [6], which has led to increased efforts to reduce its mortality through prevention or treatment. However, the impact of BC treatment on the overall healthcare budget can be dramatically high because of the costs of medications such as targeted therapy with monoclonal antibodies (trastuzumab); even though the proportion of BC patients with HER2-positive who need to use trastuzumab is approximately 20–25% [7]. To the best of our knowledge, there is no country-specific cost analysis in Iran that has reported results that can be extrapolated to the general Iranian population. Therefore, in this study we aimed at exploring how data-mining using a claims database can yield valuable information (stage-specific resource use and costs related to different stages of HER2-positive BC) for policymaking and cost-effectiveness analyses. This aim was achieved by using an example involving trastuzumab.

## Methods

### General study design

**Fig 1:**
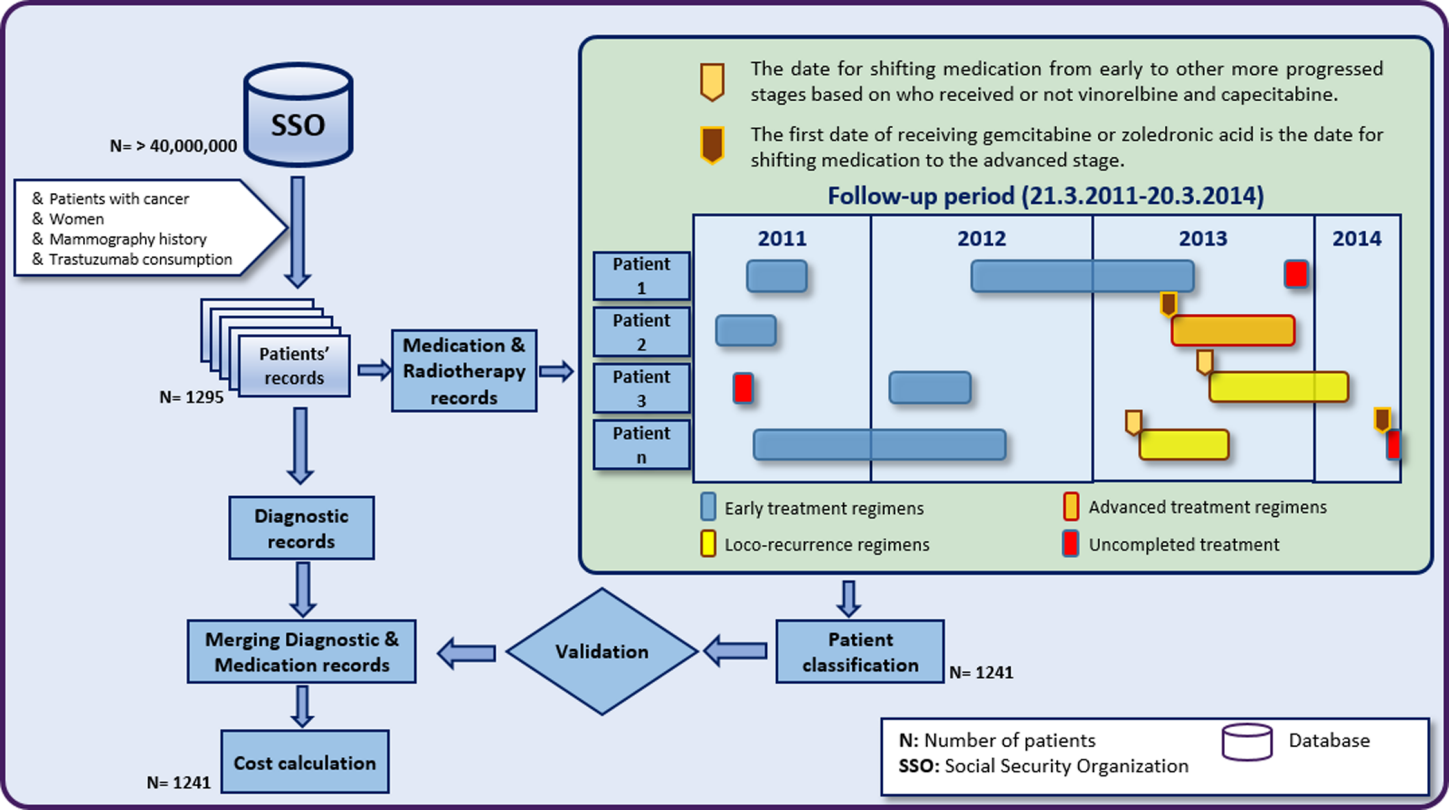
An overview of the different steps of the study.

Data from a claims database of the largest health insurance database in Iran were processed using data-mining, combined with data from other sources, and then analyzed to estimate the direct healthcare costs of HER2-positive BC in each disease stage from a payer’s perspective. The results of the data-mining process were verified using patient level-data. To access the above data sources, a research proposal was sent to the Social Security Organization (SSO) and the Cancer Research Center (CRC) to obtain claims data and patient-level data, respectively. After approval, we received the data requested in our proposal. The following sections describe the main parts of the study. Fig 1 shows an overview of the different steps of the study.

### Data sources for healthcare resource use

Three sources were used to estimate healthcare resource use and costs. To start with, most data on outpatient services and costs were drawn from a major claims database of the Social Security Organization (SSO), a social and health insurance organization that covers more than 50% (~40 million insurees) of the whole Iranian population [8]. Second, due to the absence of an integrated country level database, data on inpatient services came from two general referral hospitals (Fayazbakhsh and Alborz), which operate under the supervision of the SSO [9]. Outpatient and inpatient data were received anonymously. Lastly, we used the results of a survey of 52 oncologists [7] to estimate some minor outpatient costs in those rare cases where health care services were not covered by the SSO (e.g., services provided by doctors in their private offices). Data from these three sources were combined and analyzed in three general steps.

### Step 1: Data-mining (Identification and categorization of patients and treatment regimens)

This step comprised several sub-steps which are as follows:

#### Step 1.1: Design of patient classification algorithm

The results of a survey of oncologists [6] and other sources such as clinical guidelines [1,9,10] were used to design a strategy for patient classification. The strategy was assessed by two academic oncologists and modified based on their feedback. The final patient classification algorithm was based on medication patterns (combination and/or sequence of drug administration) seen between three index dates.

#### Step 1.2: Data requirements

Since different types of data were needed to classify patients and estimate stage-specific costs, we prepared a list of necessary data in each category and then defined our data extraction strategy.

#### Step 1.3: Data extraction

Four filters were used to select patients with HER2-positive BC in the main SSO database: diagnosis of cancer, female, history of a mammography and trastuzumab use in the past two years. The Iranian national health services coding system was used to identify women who underwent a mammography and received trastuzumab. After identifying patients, all cost data associated with BC in various healthcare services (e.g. medication, diagnosis tests, medical imaging, radiotherapy and hospitalization) were then extracted from different databases.

#### Step 1.4: Data cleaning

This step involved a check for duplications, completeness of data, and consistency of chemotherapy approaches. Any duplicated records were removed from the database. Then, to improve completeness and uniformity of data, two exclusion criteria were applied. Firstly, we excluded patients who had too few (n<6) “healthcare request sheets” over a period longer than two months since the date of the first prescription containing chemotherapy. It is worth nothing that these healthcare request sheets are used by doctors to prescribe necessary healthcare services for their patients. The Persian term for these sheets is “nos’kheh”, which we have translated to “healthcare request sheet” for lack of a better word. Each healthcare request sheet may contain one or multiple services. This is an instrument to connect doctors, other health care providers, and payers. We excluded patients with 5 or fewer healthcare request sheets because they had probably not yet completed the entire treatment regimen (type II right censoring). That is, patients who have completed the entire regimen should have at least six healthcare request sheets, which would consist of 3–4 healthcare request sheets for chemotherapy, 1–2 sheets for other medications such as hormone therapy, one sheet for laboratory test and one sheet for medical imaging during first two months of treatment onset. In fact, this threshold of 5 healthcare request sheets lies well below the number of prescriptions associated with an entire regimen. Secondly, patients were also excluded if they had a treatment regimen that was different than our previously defined treatment strategies.

#### Step 1.5: Applying the classification algorithm

**Fig 2:**
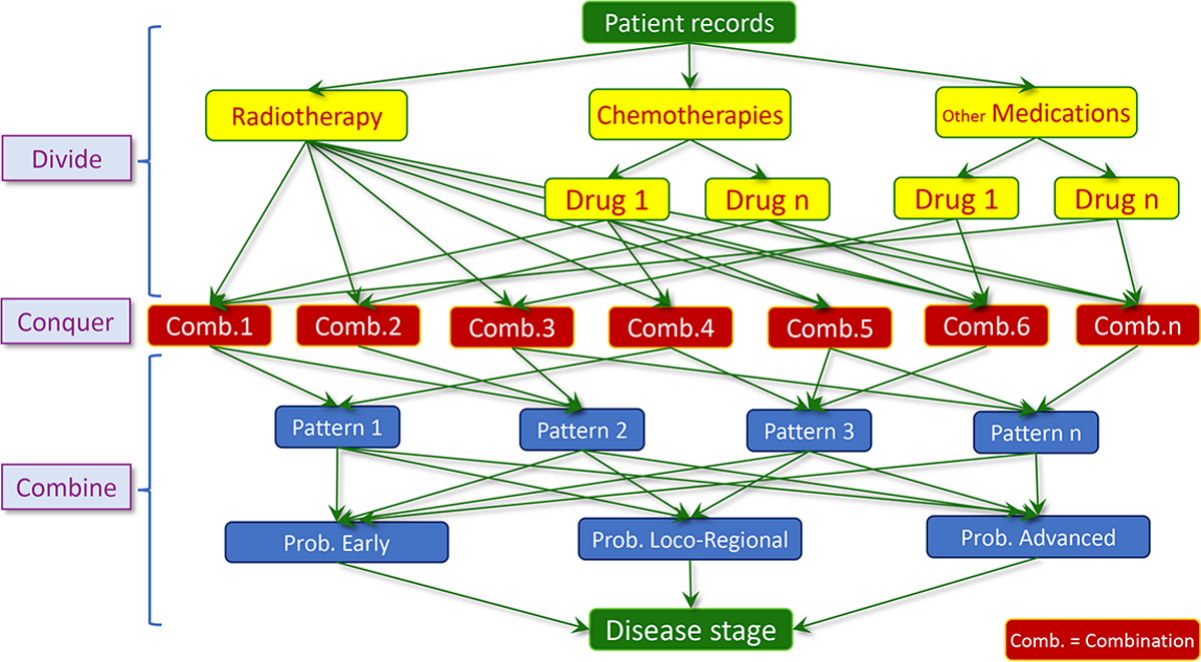
The processes of patient classification.

Classification algorithm was used to categorize patients into three different stages (“early BC”, “loco-regional BC” and “advanced BC”) based on medication patterns over a 3-year observation period (21.3.2011–20.3.2014). A decision tree induction using a divide-and-conquer approach [10] was used to predict disease stages. We also used distance matrix analysis [11,12] to obtain possible medication combinations (Fig 2). In the divide phase, we divided outpatient records into three main groups (radiotherapy, chemotherapy, and other medications). The main groups were continually divided into a number of subgroups of the same (or related) type until these subgroups became simple enough to find possible combinations by using distance matrix analysis. Then, in the conquer phase we found possible combinations of medication and radiotherapy over the observation period. Based on the results of the survey and guidelines we combined these different combinations to find the possible medication patterns. Afterwards, we calculated the probability that a particular woman had early BC, loco-regional BC or advanced BC. The probability that a woman was in a given health state was based on the ‘confidence’ about the association between our previously defined patterns and discovered patterns. Therefore, the confidence was calculated by dividing the number of discovered medication patterns representing any specific health state by the number of all discovered medication patterns in all health states [11]. For example, if we discovered three medication patterns in a patient’s profile, the value of confidence to classify that patient as being in early BC would be 33.3% (100%/3), if one of the three discovered medication patterns was only seen in early BC cases and the two other patterns in later-stage BC cases. We also defined a minimum threshold of 70% for stage-specific confidence. If the confidence of one of the three stages was higher than 70%, the patient was categorized into that stage. Otherwise, we examined the possibility of overlap between the groups. In other words, the possibility that patients progressed (e.g. from early BC to loco-regional or advanced BC) during the observation period. In this case, we looked at the difference between index dates of each health state. If the difference between two or three index dates was more than 3 months, patients were categorized into multiple stages with different index dates, otherwise the health state with the highest confidence was selected. Based on the results of a clinician survey [7], the index date for each patient with early BC was based on the first date of chemotherapy or radiotherapy (whichever happened earlier) according to possible drug combinations for early BC treatment. The loco-regional BC index date was defined as the first date when the patient received vinorelbine or capecitabine use or single-agent chemotherapy by taxols (docetaxel or paclitaxel). Finally, the index date for advanced BC patients was the first date of gemcitabine, zoledronic acid or temozolomide use. Patients who could not be classified into one of the three stages due to lack of chemotherapy records were categorized as unclassifiable.

In general, data-mining resulted in the classification of patients into three health states, including a patient-specific time interval representing the start and end dates of treatments at any particular health state.

### Step 2: Verification of patient classification algorithm

Patient-level data from the CRC were used to verify the results of the patient classification algorithm. In total, there were 51 patients at this center who were HER2-positive and SSO beneficiaries; HER2-positive BC was based on whether an IHC result showed two or more pluses or the FISH test was positive. Verification involved comparing the results of the algorithm results with the clinical results based on the tumor, node, and metastases (TNM) classification system. We examined if patients categorized as having early stage BC by the classification algorithm were in stages I or II of BC, if patients with loco-regional BC had stage III BC and if patients with advanced BC had stage IV BC. The SSO’s ID of patients in the CRC were used to link this small dataset to the claims database by SSO’s database management staff. The result of any patient classification by data-mining was approved (based on expert opinion), firstly, if the same stage had been reported in the patient dossier. Secondly, if the stage-specific index date obtained from data-mining was within two months greater than the stage-specific diagnosis date in the patient dossier.

### Step 3: Cost calculations

A national coding system covers both inpatient and outpatient healthcare services in Iran. Each healthcare service (e.g. a tablet of acetaminophen or open-heart operation) has a unique code. The national coding system is developed by the Ministry of Health. Healthcare providers must use this system (drugs + other services) when they seek payment for the procedures and services they have provided to individual patients. Healthcare providers send a text file including the patient’s ID, national codes of all services used for that patient, doctor’s ID and costs (co-payments plus payers’ share costs) to the SSO (i.e., the health insurer), either daily or monthly. Ultimately, knowing the code of any services make it possible to identify users, prescribers and the costs of that particular healthcare service.

Considering the above information, firstly, ‘service-specific costs’ were estimated using the national coding system. Outpatient care costs were calculated based on prevalent healthcare services (e.g. chemotherapy, hormone therapy, radiotherapy, diagnostic tests and medical imaging). Inpatient costs were estimated by extracting data from two hospitals on the surgical procedures used in BC management (e.g., lumpectomy, mastectomy and oophorectomy); this data were extracted from the same period as the outpatient data (21.3.2011–20.3.2014).

Afterwards, we used the results of data-mining to calculate ‘stage-specific costs’. As mentioned earlier, data-mining was used to categorize patients into three health states. Moreover, data-mining helped us to determine the duration of time that individual patients spent in each health state (stage-specific time interval). Therefore, stage-specific costs over time were estimated as follows. Costs of various outpatient services (e.g. medications, radiotherapy, diagnostic tests, medical imaging) were calculated based on the individual stage-specific time interval. For those patients whose outpatient and inpatient data were available, inpatient costs were categorized based on their stage-specific time interval obtained from data-mining on outpatient data.

In both service- and stage-specific costs, we also included treatment costs in the private sector, since the unit costs can differ between the public and private sector and patients are free to choose their healthcare provider [9,13]. Therefore, to estimate the treatment costs of patients in the private sector, the prices of healthcare services in the public sector were first converted to private prices based on the official private tariffs in the period of 2011–2014 [14,15]. Treatment costs in the private sector were then calculated based on resource use and converted prices. In certain rare instances, this conversion was also based on the results of a survey of Iranian oncologists [7]. In all above steps, costs unassociated with BC as well as the costs of uncompleted treatment regimens were excluded from the analysis.

### Analysis

The claims data were analyzed to determine the mean and distribution of resource use and costs of different medical treatment regimens, including chemotherapy, hormone therapy, radiotherapy, laboratory tests, medical imaging services and surgery in the different BC stages. 95% confidence intervals (CIs) were calculated using non-parametric bootstrapping. The number of iterations was 2000 times in all cases. All data-mining process and calculations were made using R software (version 3.2.2) for Windows [16].

## Results

### Data extraction, cleaning and patient classification

The data extraction process yielded 1,295 patients with an average age [SD] of 44 [10] years (Table 1). 54 patients were excluded from the study due to small number of prescriptions or inconsistency between their chemotherapy approach and our previously defined drug combinations. Of the 1,241 patients, 802, 125 and 218 patients were categorized into early BC, loco-regional BC and advanced BC, respectively during a mean [SD] follow-up period of 679 [289] days. The mean ages of women in the different health stages ranged from 45–48 years and average follow-up durations per patient [SD] was 425 [210] days. Furthermore, on average, the mean follow-up durations per stage [SD] were 397 [198] (early BC), 349 [234] (loco-regional BC) and 318 [204] (advanced BC) days. During the follow-up period, 158 patients with early BC progressed to loco-regional BC (n = 41) or advanced BC (n = 106), 19 patients progressed from loco-regional BC to advanced BC, and 11 patients progressed through all three BC stages. Finally, 284 patients were not classifiable due to lack of chemotherapy records.

**Table 1:** Age characteristics of study patients with HER2-positive BC.

Stages	Before analysis	Early BC	Loco-regional BC	Advanced BC
Number of patients	1295	802	125	218
**Age**	at start date (2011-03-21)	at index date	at index date	at index date
Average [SD]	44 [10]	45 [10]	46 [10]	48 [10]
Age range (%)				
≤34	222 (17)	120 (15)	15 (12)	21 (10)
35–44	447 (35)	261 (33)	46 (37)	54 (25)
45–54	409 (32)	267 (33)	38 (30)	84 (39)
55–64	172 (13)	124 (15)	22 (18)	47 (22)
≥65	45 (3)	30 (4)	4 (3)	12 (6)

**Abbreviations: BC:** Breast cancer; **SD**: Standard deviation.

### Patient classification verification

Verification of patient classification was based on data obtained from CRC on a group of 51 patients; 65% (n = 33) were in early BC, 10%(n = 5) in loco-regional BC and 25%(n = 13) in advanced BC. Two (4%) patients had both loco-regional and advanced BC in the observation period. Some patients with loco-regional and advanced BC were incorrectly categorized as having early stage BC; these represented 14% (n = 7) of patients in the loco-regional stage and 2% (n = 1) of patients with advanced BC). Therefore, the accuracy by stage was 100% (25/25) for early BC, 46% (6/13) for loco-regional BC and 93% (14/15) for advanced BC. In general, verification of the classifications based on data-mining revealed an 85% accuracy rate.

### Service-specific costs—Outpatient costs

The different outpatient costs are shown in Table 2. The costs were weighted based on proportion of resource use between public and private sectors; medication costs required no weighting because there are no price differences between the two sectors. The costs also included the costs of drugs used as concomitant drugs like antiemetics. The most common chemotherapy regimen among early BC patients consisted of a taxane (docetaxel or paclitaxel) along with doxorubicin and cyclophosphamide (66%). The more expensive medications in early BC comprised different combinations of chemotherapy including docetaxel. Patients with loco-regional BC were almost evenly distributed among different treatment regimens. Vinorelbine was used in the treatment of almost half (45%) of the advanced BC patients. Tamoxifen and letrozole were the two most common types of hormone therapy.

**Table 2:** Average costs of outpatient services.

Costs as of 20.03.2014 (1€ = 34,000 rials)	Provided in the public sector*	n = 1,241		
		Treated (%)	Mean [SD] €	Range €
**Medications**				
**Chemotherapy (Early BC)**				
*All*	NA	802 (65)	11,865 [8,247]	623–45,275
*trastuzumab*	NA	757 (61)	10,565 [7,668]	332–40,447
*doxorubicin + cyclophosphamide + docetaxel*	NA	314 (25)	2,480 [1,335]	105–6,925
*doxorubicin + cyclophosphamide + paclitaxel*	NA	236 (23)	1,164 [433]	249–2,444
*epirubicin + cyclophosphamide + docetaxel*	NA	100 (8)	2,507 [1,16]	703–5,459
*fluorouracil + epirubicin + cyclophosphamide + docetaxel*	NA	65 (5)	2,108 [988]	372–4,737
*epirubicin + cyclophosphamide + paclitaxel*	NA	39 (3)	1,321 [390]	638–2,249
*fluorouracil + doxorubicin + cyclophosphamide + docetaxel*	NA	19 (2)	1,747 [802]	713–3,474
*paclitaxel OR docetaxel + carboplatin*	NA	14 (1)	1,953 [1,081]	297–3,721
*fluorouracil + epirubicin + cyclophosphamide + paclitaxel*	NA	12 (1)	940 [235]	604–1,337
*fluorouracil + doxorubicin + cyclophosphamide + paclitaxel*	NA	7 (1)	1,293 [560]	402–2,196
**Chemotherapy (Loco-regional BC)**				
*All*	NA	125 (10)	7,776	75–34,363
*trastuzumab*	NA	90 (9)	8,346	820–29,700
*capecitabine*	NA	42 (3)	604	140–2,165
*vinorelbine*	NA	26 (3)	1,129	26–4,929
*paclitaxel*	NA	19 (2)	750	188–2,846
*paclitaxel OR docetaxel + capecitabine*	NA	18 (2)	1,881	151–4,884
*docetaxel*	NA	18 (2)	1,741	392–3,697
*paclitaxel OR docetaxel + carboplatin*	NA	5 (0)	1,597	446–4,047
**Chemotherapy (Advanced BC)**				
*All*	NA	218 (18)	14,191 [12,045]	108–49,453
*trastuzumab*	NA	198 (16)	11,471 [8,793]	756–42,560
*vinorelbine*	NA	72 (6)	1,967 [1,769]	41–8,845
*capecitabine*	NA	63 (5)	1,277 [845]	159–3,579
*paclitaxel OR docetaxel + carboplatin*	NA	15 (1)	1,672 [1,380]	236–5,627
*paclitaxel OR docetaxel + capecitabine*	NA	29 (2)	1,347 [1,080]	93–2,665
*docetaxel*	NA	16 (1)	1,039 [830]	265–2,690
*paclitaxel*	NA	13 (1)	2,021 [1,918]	155–6,217
*doxorubicin + cyclophosphamide + docetaxel*	NA	3 (0)	1,656 [839]	693–2,227
*doxorubicin + cyclophosphamide + paclitaxel*	NA	3 (0)	1,207 [685]	542–1,910
*fluorouracil + epirubicin + cyclophosphamide + paclitaxel*	NA	3 (0)	1,839 [640]	1,223–2,501
*epirubicin + cyclophosphamide + docetaxel*	NA	1 (0)	713 [NA]	713–713
*fluorouracil + epirubicin + cyclophosphamide + docetaxel*	NA	1 (0)	1,683 [NA]	1,683–1,683
*fluorouracil + doxorubicin + cyclophosphamide + paclitaxel*	NA	1 (0)	2,529 [NA]	2,529–2,529
*epirubicin + cyclophosphamide + paclitaxel*	NA	0 (0)	0	0–0
*fluorouracil + doxorubicin + cyclophosphamide + docetaxel*	NA	0 (0)	0	0–0
*lapatinib + capecitabine*	NA	0 (0)	0	0–0
**Hormone therapy**				
*All*	NA	662 (53)	168 [241]	1–1,257
*tamoxifen*	NA	272 (22)	11 [8]	0–42
*tamoxifen + triptorelin*	NA	135 (11)	407 [245]	51–1,145
*letrozole*	NA	115 (9)	67 [48]	8–327
*triptorelin*	NA	56 (5)	259 [206]	41–812
*tamoxifen + letrozole + triptorelin*	NA	27 (2)	475 [329]	69–1,257
*tamoxifen + letrozole*	NA	26 (2)	75 [44]	22–169
*exemestan*	NA	9 (1)	436 [178]	164–777
*letrozole + triptorelin*	NA	7 (1)	553 [356]	229–1,187
*tamoxifen + exemestan*	NA	2 (0)	553 [232]	389–717
*goserelin*	NA	0 (0)	0	0–0
**Visit and administration**				
*Oncologist visits per session*	0.54^#^	1,241 (100)	4^+^ [2]	2^Y^-7 ^Y^
*Chemotherapy administration per session*	0.61^#^	1,241 (100)	28^#^ [23]	4^#^-103^#^
**Cardiac monitoring services**				
*Cardiac drugs*	NA	676 (55)	8 [15]	1–156
*Internist or Cardiologist visits*	0.45	715 (70)	16 [21]	3–206
*Cardiology services*	0.63	115 (9)	518 [457]	136–1,989
**Radiotherapy**	0.31	231 (19)	2,426 [1,313]	110–8,530
**Laboratory tests**				
*All*	0.21	1,180 (97)	112 [88]	2–1,308
*Sampling fees*	0.26	1,151 (93)	5 [4]	0–33
*Hematology*	0.25	1,129 (91)	10 [7]	1–50
*Clinical chemistry*	0.23	1,060 (85)	24 [24]	1–226
*Cytopathology*	0.19	786 (63)	6 [8]	2–56
*Descriptive pathology*	0.19	741 (60)	53 [34]	5–352
*Tumor marker*	0.16	693 (56)	32 [28]	0–263
*Urinalysis*	0.22	582 (47)	1 [1]	0–13
*Hormones*	0.15	549 (44)	13 [12]	3–105
*Serology and immunology*	0.17	403 (32)	10 [15]	1–208
*Microbiology*	0.27	332 (27)	2 [2]	0–24
*Specific clinical chemistry*	0.17	320 (26)	8 [6]	1–43
*Coagulation*	0.17	241 (19)	3 [3]	1–36
*Cytogenetics*	0.17	24 (2)	162 [114]	2–400
*Molecular genetics*	0.06	21 (2)	87 [244]	16–1,140
**Medical imaging**				
*All*	0.19	1,157(93)	612 [1,158]	3–8,697
*Ultrasound*	0.14	833 (67)	29 [26]	4–197
*X-ray*	0.22	787 (63)	25 [18]	3–115
*CT Scan*	0.24	666 (54)	73 [58]	12–443
*Nuclear medicine*	0.15	476 (38)	73 [66]	15–1,347
*MRI*	0.22	313 (25)	80 [67]	30–715
*Other services*	0.25	26 (2)	83 [27]	21–115

**Abbreviations: BC:** Breast cancer; **NA:** Not applicable; **SD**: Standard deviation

* This column shows the proportions of healthcare utilization in the public sector. (Number of services in public sector/Total number of services (private + public).

# The data obtained from the results of a questionnaire survey. [7]

Y The unit costs obtained from the national health care services tariff 2014 [15]

+ The unit cost obtained from the SSO yearly report (2014) [13]

**Fig 3:**
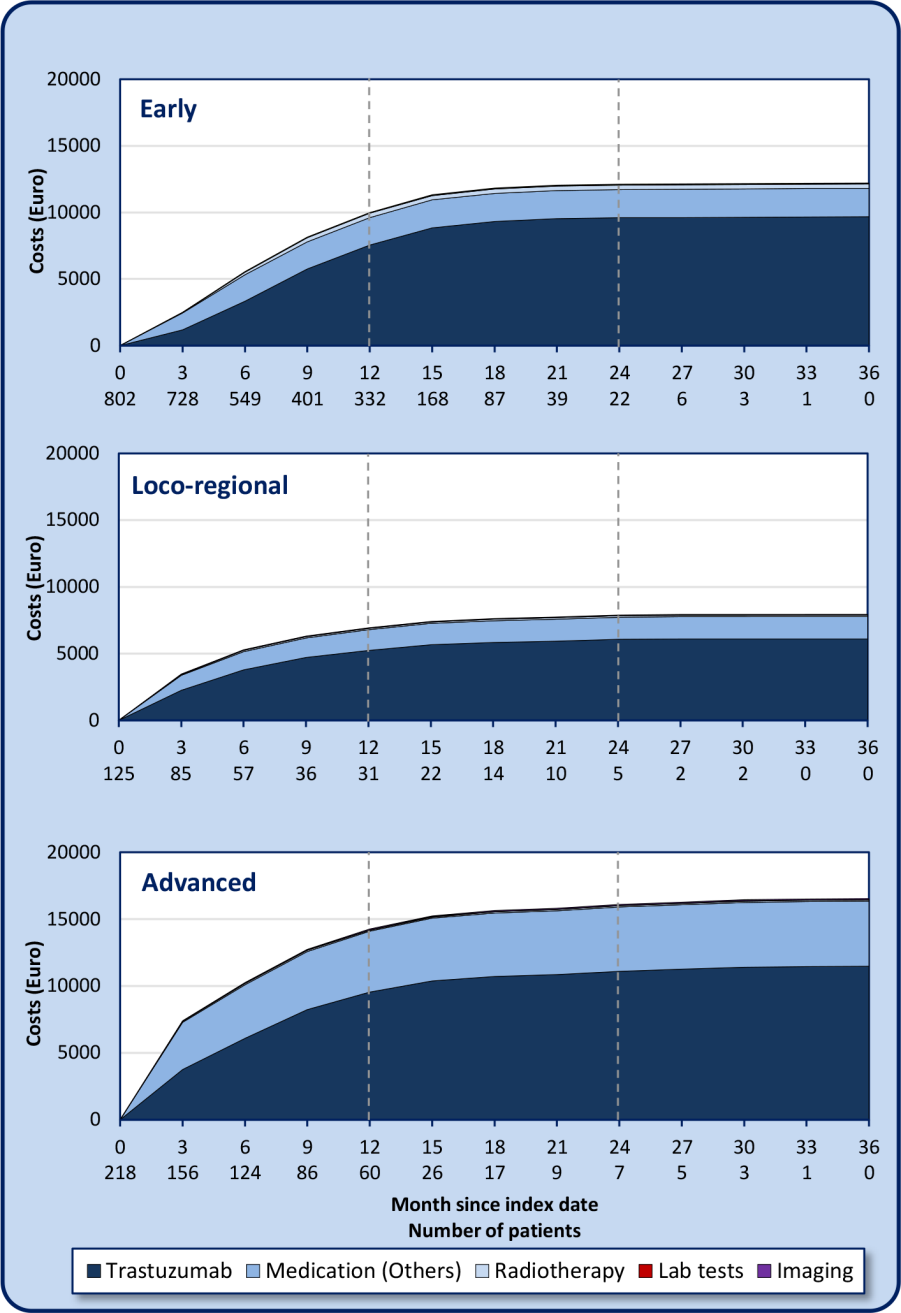
Cumulative outpatient main healthcare care costs since the index dates in different health states of HER2-positive breast cancer in Iran, during a 3-year follow-up period (21.3.2011–20.3.2014).

In all three BC stages, there is a rapid increase in outpatient-related costs during the first year after the index date (Fig 3), followed by a gradual increase and a plateau.

### Service-specific costs—Inpatient costs

Table 3 shows the inpatient costs based on the two main surgical operations (mastectomy and oophorectomy), which were based on information from two hospitals and 710 patients during a 3-year follow-up period (21.3.2011–20.3.2014). The mean costs were calculated using all components of hospitalization costs, including procedure, nursing, accommodation, medication, laboratory tests, medical imaging, pathology, medical equipment and physiotherapy. The mean [SD] costs of mastectomy and oophorectomy were 293 [178] and 261 [172] euros, respectively.

**Table 3:** Average costs of mastectomy and oophorectomy. (Costs as of 20.03.2014; 1€ = 34,000 rials).

Inpatient services	Number of patients	Mean admission days	Mean cost €	Range €
**Mastectomy**	**141**	**3.57 [3.09]**	**293 [178]**	**31–1,242**
*Partial*	42	1.93 [1.47]	125 [138]	38–718
*Partial with axillary lymphadenectomy*	7	3.29 [1.11]	179 [63]	103–254
*Simple*, *complete*	20	1.60 [1.43]	103 [65]	53–350
*Radical*, *including pectoral muscles and axillary lymph nodes*	7	4.57 [3.26]	190 [90]	68–324
*Radical*, *including internal mammary lymph nodes*, *unilateral*	41	5.49 [3.52]	261 [174]	111–1,144
*Modified radical*, *including axillary lymph nodes but leaving pectoral muscles*	24	4.63 [2.90]	309 [247]	31–1,242
**Oophorectomy**	**569**	**3.38 [4.68]**	**261 [172]**	**62–2,550**
*Partial resection of ovary (unilateral or bilateral)*	145	3.60 [3.62]	161 [102]	62–756
*Wedge resection or bisection of ovary (unilateral or bilateral)*	14	6.14 [7.90]	209 [111]	113–547
*Ovarian cystectomy (unilateral or bilateral)*	374	3.24 [4.83]	200 [196]	66–2,550
*Oophorectomy*, *Unilateral or bilateral*, *partial or total*	36	2.92 [1.42]	166 [104]	83–570

### Stage-specific direct healthcare costs

As Table 4 shows, trastuzumab accounted for the largest share of total direct healthcare costs; its share was almost 76% for early BC, 73% for loco-regional BC and 56% for advanced BC. The relative costs of other medications (including chemotherapy, cardiovascular drugs, analgesics and hormone therapies) were similar in early BC and loco-regional BC but much greater in advanced BC (Fig 3). Other cost components comprised a limited share of the total costs. For inpatient services, we found 12 patients who were included in both the outpatient and inpatient data. Nine of them underwent surgery during early stage BC while the others were treated during advanced BC, according to the date of surgery (Table 4).

**Table 4:** The overall average costs (public and private sectors) in three stages of BC during the follow-up period. Results after applying nonparametric bootstrapping. Costs as of 20.03.2014 (1€ = 34,000 rials).

		Early BC	Loco-regional BC	Advanced BC
Number of patients		802	125	218
Average follow up period (Days [SD])		397 [198]	349 [234]	318 [204]
	Provided in the public sector*	Mean € (SE)	Mean € (SE)	Mean € (SE)
		95%CI	95%CI	95%CI
**Trastuzumab**	**NA**	**9,018** (233)	**6,009** (591)	**12,985** (648)
		8,566–9,452	4,975–7,296	11,745–14,340
**Medications (Others)**	**NA**	**1,838** (44)	**1,642** (125)	**3,547** (127)
		1,764–1,944	1,422–1,906	3,274–3,858
**Specialists visit**	**0.54**	**79** (4)	**86** (7)	**119** (11)
		76–82	74–98	107–132
**Chemotherapy administration**^#^	**0.61**	**135** (46)	**145** (42)	**220** (16)
		122–149	129–161	201–239
**Radiotherapy**	**0.46**	**440** (32)	**243** (61)	**121** (48)
		382–507	137–385	57–298
**Laboratory tests**	**0.16**	**57** (2)	**51** (6)	**54** (5)
		54–61	42–65	46–64
**Medical imaging**	**0.24**	**55** (2)	**77** (7)	**91** (10)
		51–60	64–91	75–118
**Inpatient services**	**0.83**	**174** (49)	-	**335** (102)
		105–243		215–456
**Total** (95%CI)		**11,796**	**8,253**	**17,742**
		**9,356–12,498**	**6,843–10,002**	**15,720–19,505**

**Abbreviations: BC:** Breast cancer; **CI:** Confidence interval; **NA:** Not applicable; **SD**: Standard deviation; **SE:** Standard error

* This column shows the proportions of healthcare utilization in the public sector. (Number of services in public sector/Total number of services (private + public).

# The data obtained from the questionnaire [7]

The average cumulative direct healthcare costs for early BC over the follow-up period were €11,796 (95% CI: €9,356-€12,498). The treatment costs for loco-regional BC averaged €8,253 (95%CI: €6,843-€10,002). In contrast, the mean costs for advanced BC (95% CI) were €17,742 (€15,720-€19,505), which was roughly 50% higher than the costs for early stage BC.

## Discussion

Generally, BC is a costly illness throughout the world. The use of monoclonal antibodies like trastuzumab or pertuzumab to treat BC can have a significant budget impact, specifically in MICs. We performed a real-world cost analysis using a large claims database and a validated patient classification algorithm to estimate the stage-specific healthcare utilization and costs associated with HER2-positive BC in Iran.

Our findings show that total direct healthcare costs per patient averaged €11,796 for early BC, €8,253 for loco-regional BC and €17,742 for advanced BC during a 3-year follow-up period (21.3.2011–20.3.2014). The largest share of the total cost was spent on trastuzumab. Other medications like chemotherapy and hormone therapy were the second most important cost drivers in BC. The relative costs of other medications associated with advanced BC were dramatically higher compared with early and loco-regional BC (Fig 3). This was due to the use of other expensive medications to manage the effects of cancer metastasis to other organs or other medications to control the side-effects of chemotherapy. In early BC, the most common regimens of chemotherapy were the combination of an anthracycline antibiotic like doxorubicin and cyclophosphamide followed by a taxane-like docetaxel. This corresponds with the results of a previous study [7]. In contrast to outpatient services, inpatient healthcare cares had only a minor impact on total costs. It should be noted that BC treatment in Iran is mostly provided on an outpatient basis, something that has been reported in studies in other countries [17]. For example, Vera-Lionch et al showed that inpatient costs of BC care, even for metastatic BC, accounted for approximately 20% of total BC costs in the United States [17]. Another observation is that our results showed that the average age of BC is lower than the average age seen in other countries (Table 1). Fifty years was considered as the average age for early HER2-positive BC based on the majority of trials [18–20]. These results also correspond with the findings of previous Iranian epidemiological studies [21,22].

A previous study estimated the direct costs of treating patients with BC based on patients referred to one hospital in Iran [23]. Although that study examined the costs (US$) in different disease stages over a five-year period (2005–2010) (Stage I: (public: 4,386; private: 8,467), Stage II: (public: 4,692; private: 8,704), Stage III: (public: 6,845; private: 10,886), Stage IV: (public: 8,077; private: 8,935)) [23], its study population was small, which limited the precision of the results, and only one hospital was included, which limited the generalizability of the findings. In addition, that study did not report the costs of patient subgroups with tumor receptor expression. In contrast, our study provides more details concerning different treatment patterns and cost components on a national level, although it focused only on HER2-positive BC. Another recently published study of the economic burden of BC in Iran estimated the overall direct healthcare costs of BC (US$175,832,701) using a societal perspective [24]. However, comparisons between their results and our results are not possible since the authors did not report individual direct healthcare costs for HER2-positive BC.

Like any study, our study also has its limitations. The first limitation relates to our study population. One could argue that the claim data covers approximately 50% of the population and therefore may not represent the whole Iranian population. However, our results can be generalizable to the whole population because the four main payers in Iran have the same benefit package and because the SSO, which provided the data used in our study, covers the insurees from different socio-economic classes in all provinces [6]. A second possible limitation is that focusing on BC patients treated with trastuzumab may mean that the costs we found are not generalizable to the costs for other BC patients. While this is certainly true for trastuzumab costs, this selection is likely to have only a limited effect on other costs since trastuzumab use does not affect the choice of drug combinations or decisions about dose adjustment of chemotherapy [25,26]. Therefore, our results can reflect direct healthcare costs in both patient populations by including or excluding the costs associated with trastuzumab. Thirdly, since the SSO database did not include inpatient data, we collected inpatient data from two hospitals, which means that our cost estimates of inpatient care might not reflect the average costs across all hospitals in Iran. However, this limitation has no major impact on the overall results since BC management is mainly performed on an outpatient basis in Iran and the outpatient costs were based on data from more than 45,700 (~100%) healthcare providers [13]. Fourthly, while data-mining achieved a high accuracy rate of 85% (of classifying patients into the correct stage of BC), it was not perfect, meaning that some patients were incorrectly classified. Cost estimates using the verification results led to an increase in total costs for loco-regional BC (i.e., €10,161, 23% higher than the €8,253 from data-mining) and a slight decrease in total costs for advanced BC (i.e., €17,402, 2% lower than the €17,742 from data-mining), respectively. Therefore, total costs in loco-regional BC should be used with caution. The accuracy of classification could be improved by adding electronic results of diagnostic tests to the patient classification algorithm, if the results were available. Fifthly, since the patient population and treatment practice at the center used to validate our data-mining algorithm may be different than patients and treatments elsewhere, it is possible that the true accuracy may be different from the estimated accuracy. Finally, the cost estimates in our study do not include illegal and informal payments [27] by patients to healthcare providers. Authorities in the Ministry of Health currently claim that they were able to reduce improper payments significantly over the past three years as a result of president Rouhani’s healthcare reform implementation [28]. However, due to lack of information we were not able to determine its impact on the final results of this study. The importance of excluding these costs may not be significant if the perspective of the healthcare sector is taken when estimating costs but could be significant if a societal perspective is taken.

This study shows that if relevant data are available, data-mining techniques on claims data can provide cost estimates that are invaluable in performing real-world cost-effectiveness analyses in MICs. National level cost-effectiveness analyses can use these cost estimates to assess whether new medicines or procedures are cost-effective. For example, policymakers can reduce uncertainties about the cost-effectiveness of a monoclonal antibody like trastuzumab by performing a country-specific cost-effectiveness analysis using the results of our real-world cost analysis. Despite the advantages of using claims databases, these data sources usually do not provide clinical, laboratory, and imaging data as well as some important treatment events such drug-related adverse events.

The quality of reimbursement decision-making can be improved by using information about actual healthcare utilization and costs. One option in high-income countries would be an extensive cost analysis supported by patient registry data and electronic health records. However, implementation of comprehensive patient registries is costly and sometimes logistically infeasible in many MICs. If integrated electronic records are unavailable, researchers may have to rely on small sample sizes or expert opinion (e.g., [23], [24]) to estimate the direct healthcare costs of an illness. Both of these methods have their shortcomings, which might affect the internal validity and generalizability of the results. Claims databases would be useful sources for cost analyses in MICs as long as the disease status of patients is known or adequately predicted.

## Conclusion

This study estimated the stage-specific direct healthcare costs associated with HER2-positive BC in Iran using a large claims database and data-mining with validated patient classification algorithms. The findings show that the largest component in overall costs in all stages of BC is medication and that trastuzumab is the major cost driver. These real-world data can support cost-effectiveness analyses of implementing new technologies for HER2-positive BC management and thereby help to optimize reimbursement decision-making in an MIC.
